# Pattern Recognition via the Toll-Like Receptor System in the Human Female Genital Tract

**DOI:** 10.1155/2010/976024

**Published:** 2010-04-11

**Authors:** Kaei Nasu, Hisashi Narahara

**Affiliations:** Department of Obstetrics and Gynecology, Faculty of Medicine, Oita University, Oita 879-5593, Japan

## Abstract

The mucosal surface of the female genital tract is a complex biosystem, which provides a barrier against the outside world and participates in both innate and acquired immune defense systems. This mucosal compartment has adapted to a dynamic, non-sterile environment challenged by a variety of antigenic/inflammatory stimuli associated with sexual intercourse and endogenous vaginal microbiota. Rapid innate immune defenses against microbial infection usually involve the recognition of invading pathogens by specific pattern-recognition receptors recently attributed to the family of Toll-like receptors (TLRs). TLRs recognize conserved pathogen-associated molecular patterns (PAMPs) synthesized by microorganisms including bacteria, fungi, parasites, and viruses as well as endogenous ligands associated with cell damage. Members of the TLR family, which includes 10 human TLRs identified to date, recognize distinct PAMPs produced by various bacterial, fungal, and viral pathogens. The available literature regarding the innate immune system of the female genital tract during human reproductive processes was reviewed in order to identify studies specifically related to the expression and function of TLRs under normal as well as pathological conditions. Increased understanding of these molecules may provide insight into site-specific immunoregulatory mechanisms in the female reproductive tract.

## 1. Introduction

The mucosal surface of the respiratory, gastrointestinal, and urogenital tracts separates the external environment from the internal sterile environment and thus represents the first line of defense against microbes. These mucosal innate systems consist principally of mechanical, chemical, and cellular components. The first of these, the mechanical component, primarily carries out the physical barrier function of the mucosa, but also includes physiological functions such as cilial action, motility, desquamation, and mucous secretion. The second component, the chemical component, can be further divided into three subcomponents: soluble or cell-associated pattern recognition molecules, proteins, and peptides, which are responsible for orchestration of the immune response. The third component of the innate immune system is the cellular component, which includes epithelial cells, stromal fibroblasts, and various inflammatory leukocytes. 

Mucosal epithelial cells constitute a crucial part of the innate immune system and are actively engaged in the first line of defense against microbial infections. Defense at the epithelial barrier includes the mechanical aspect of preventing penetration of the structure by microorganisms. The mucosal epithelial cells are known to function as sentinels that recognize antigens, and they respond in a manner leading to bacterial and viral eradication, as well as send signals to underlying immune cells. When a pathogenic challenge exceeds the protective capacity of the mucosal epithelial cells, they trigger a series of alarm signals resulting in the secretion of chemokines ultimately important for the recruitment of other components of the innate defense network, which in turn leads to the development of an acute inflammatory reaction. Binding of a pathogen with the epithelium can lead to cell death by necrosis, apoptosis, or internalization of the organism; however, the invading organism may remain on the cell surface and induce disease from this location. At this stage, increased vascular permeability leads to extravasation of acute-phase proteins and protein complement into the damaged tissue. Also affected are the endothelial adhesion molecules that reduce the activity of phagocytic granulocytes, allowing them to leave blood vessels and be transported along a chemotactic gradient towards the pathogen. These various steps, which run parallel rather than in a sequential manner, are controlled by cytokines secreted from the cellular components (e.g., epithelial cells and inflammatory leukocytes) of the innate system. While these immediate mechanisms are in progress, antigenic material is processed by dendritic cells and macrophages for presentation to T cells, a process which represents the initiation of the more slowly developing acquired responses. However, the mechanisms leading to these latter responses differ in the mucosal linings of different organs [1]. 

The major purpose of the innate immune system is to react rapidly to infectious agents with the initiation of an inflammatory response, and to form subsequent adaptive immune responses. After a pathogen makes contact with the epithelial surface, signals are generated that result in the production of chemokines, cytokines, prostaglandins, and leucotrienes by the epithelium, signaling cell injury [2]. However, the pathogen also interacts with other components of the innate immune system such as dendritic cells and macrophages. The basis of this activation of the innate immune system is pattern recognition [3]. Pathogens are characterized by specific arrangements of key molecules called pathogen-associated molecular patterns (PAMPs) and are recognized by pattern recognition receptors (PRRs). The PAMPs are vital structures of the microbial cell that have altered little over evolutionary time spans and include lipopolysaccharide (LPS), lipoproteins, peptidoglycan (PGN), lipoarabinomannan, and oligosaccharides. The PRRs are found on many cells of the innate immune system including epithelial cells, fibroblasts, and inflammatory leukocytes. There are several different families of PRRs such as scavenger receptors, Toll-like receptors (TLRs), nucleotide binding oligomerization domain- (NOD-) like receptors, retinoic-acid-inducible protein (RIG)-I-like receptors, formyl peptide receptors, mannose and glycan receptors, C-type lectin receptors, complement receptors, and CD14. Among the PRRs, TLRs are capable of sensing organisms ranging from bacteria to fungi, protozoa, and viruses, and they play a major role in innate immunity.

## 2. Structure and Characteristics of Human Female Genital Mucosa

**Figure 1:**
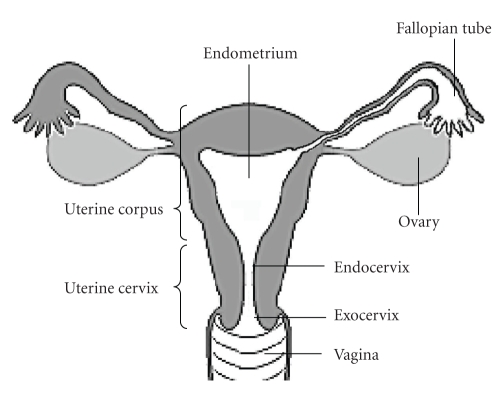
Structure of the female genital tract.

The female genital tract is composed of a sequence of cavities. The external genital tract at the vulva leads into the vagina, which connects in succession to the uterine cervix, the endometrium, and then to the fallopian tubes (Figure 1). The lumen of the lower genital tract (vagina and ectocervix) is lined with squamous epithelium; whereas the upper genital tract (endocervix, endometrium, and fallopian tubes) is lined with columnar epithelium. The surface epithelium serves a critical function, that is, as the defensive front line of the mucosal innate immune system in the female genital tract. Under normal conditions, the mucosa of the female genital tract appears to be in a state of controlled inflammation. 

The upper genital tract is virtually free of organisms, with little commensal microbial activity [4]. Recent evidences suggest that there is a site-specific mucosal immune system in the female upper genital tract, including the endometrium and fallopian tubes, which differs from that described for the intestinal, respiratory, and lower genital tracts. This putative immune system in the fallopian tubes and endometrium might contribute to the maintenance of an aseptic milieu, free of the microorganisms that sporadically colonize the upper genital tract. It is essential that the mucosal epithelium of the upper genital tract has the capacity to recognize and respond to ascending pathogens, while at the same time avoiding a state of chronic inflammation that might disrupt the epithelial barrier. The upper genital tract is vulnerable to the spread of microorganisms from the lower genital tract, resulting in the development of infectious diseases such as endometritis and salpingitis [5]. The sequelae of such chronic inflammation of the female genital tract would be highly detrimental to the host and would include increased transmission of sexually transmitted diseases [6].

 The cervical and vaginal epithelium is constantly exposed to microorganisms including species of commensal as well as pathogenic organisms; as anaerobic bacterial flora is normally present in the vagina. Therefore, the mucosal surface of the lower genital tract represents a complex biosystem that provides a barrier against the outside world and participates in both innate and acquired immune defense systems. This mucosal component has adapted to a dynamic, nonsterile environment challenged by a variety of antigenic/inflammatory stimuli associated with sexual intercourse and endogenous vaginal microbiota. The cervicovaginal epithelial cells that line the mucosal surface are often the first cells to come into contact with microbial pathogens; normally there are very few immune cells that present in the cervicovaginal mucosa and lumen [6, 7]. The cervicovaginal epithelial cells initiate and coordinate the inflammatory response, altering the adjacent epithelium and the underlying stromal fibroblasts and immune cells to counter the potential danger posed by various microorganisms.

## 3. Pattern Recognition via the TLR System in Humans

Rapid innate immune defenses against microbial infection usually involve the recognition of invading pathogens by specific PRRs recently attributed to the family of TLRs. TLRs are present in plants, invertebrates, and vertebrates and they represent a primitive host defense mechanism against microorganisms [8–11]. As shown in Table 1, TLRs recognize conserved PAMPs synthesized by microorganisms including bacteria, fungi, parasites, and viruses as well as endogenous ligands associated with cell damage, such as heat-shock protein 60, heat-shock protein 70, polysaccharide fragments of heparin sulfate, hyaluronic acid, fibrinogen, fibronectin DA domain, and mRNA [12]. Members of the TLR family include 10 TLRs identified in humans thus far, which recognize distinct PAMPs produced by various bacterial, fungal, and viral pathogens. The recognition of bacterial PAMPs (e.g., LPS, PGN, flagellin) is mediated by TLR1, 2, 4, 5, and 6 [13–16]. Among these TLRs, four are designed to recognize nucleic acids: TLR3, TLR7, TLR8, and TLR9 [17–20]. TLR7 and TLR8 recognize nucleotide derivatives, such as self and viral single-stranded RNA [19, 20], and TLR9 binds unmethylated DNA found in bacteria [17]. In contrast, TLR3 recognizes double-stranded RNA (dsRNA) [18], a molecular signature of RNA viruses [21]. Therefore, it is likely that TLR3 plays a physiological role in antiviral innate immunity [22]. 

**Figure 2:**
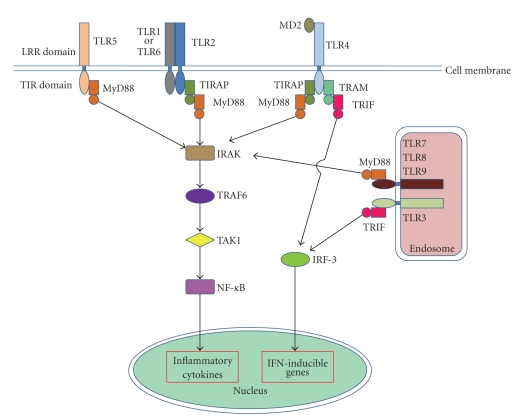
Simplified diagram of TLR signaling pathways.

TLRs are transmembrane signaling proteins that are designed to recognize, with high specificity, various proteins, lipids, carbohydrates, and nucleic acids of invading microorganisms. In turn, TLRs activate signaling cascades in cells that can trigger immune and inflammatory responses to combat the infectious agent [3, 23, 24]. Although every member of the TLR family responds to a specific ligand, they all share strong similarities in terms of their structures and properties [25]. TLR proteins are located on either the plasma membrane or internal membranes. Their cytoplasmic signaling domain is separated by a single membrane-spanning domain from the ligand-recognizing extracellular or luminal domain, which contains multiple repeats of a leucine-rich repeats (LRRs) motif XXLXLXX. The 19–25 tandem copies of LRRs are thought to provide a highly specific binding surface for the cognate ligand. The cytoplasmic domain of the TLR family shares extensive homology with that of the interleukin (IL)-1 receptor family and is referred to as the toll-IL receptor (TIR) domain, which extends to about 200 residues [26, 27]. Ligand binding to TLRs leads to a common signal transduction pathway involving TIR, which couples with adaptor molecules including MyD88 [28] that binds to TLR1, TLR2, TLR4, TLR5, TLR6, TLR7, TLR8, TLR9, and TLR10 [29–31]; MAL/TIRAP, a MyD88 homologue that binds to TLR1, TLR2, TLR4, and TLR6 [32, 33]; TRAM that binds to TLR4; TRIF/TICAM1 that binds to TLR3 and TLR4 [34] (Figure 2) [28, 29]. There are two main pathways activated by the TLR family, the MAL/MyD88-dependent and the MyD88-independent TRAM/TRIF pathway. Signaling through MyD88 activates nuclear factor (NF)-*κ*B and induces many cytokines including tumor necrosis factor (TNF)-*α* and IL-6. Stimulation of TRIF signaling pathway activates the interferon (IFN) regulatory factor (IRF) family to induce production of type I IFNs [35, 36]. Subsequently, a signaling complex is formed that includes the IL-1 receptor-associated kinases (IRAKs), Tollip and TNF receptor-associated factor 6 (TRAF-6), transforming growth factor (TGF)-*β*-activated kinase (TAK1), and the TAK1 binding proteins TAB1 and TAB2. The formation of this complex ultimately results in the phosphorylation of the inhibitor of NF-*κ*B (I*κ*B) and the activation of the NF-*κ*B pathway [30, 31, 37]. In this manner, TLRs regulate a number of consequences such the production of proinflammatory cytokines, the upregulation of costimulatory molecules on antigen-presenting cells, and the maturation of naive dendritic cells. TLR binding to microbial ligands is thus a key step in the acute inflammatory response. 

It should be noted that cell surface TLRs (TLR1, TLR2, TLR4, TLR5, and TLR6) appear to recognize microbial products such as LPS or lipopeptides; whereas intracellular TLRs (TLR3, TLR7, TLR8, and TLR9) recognize nucleic acids [28, 29]. The availability of endogenous ligands and the amount of cell-surface TLRs are both tightly restricted to maintain sufficient TLR responses for the containment of pathogens, without inducing detrimental responses in the host. All of the nucleic acid-recognizing TLRs are expressed on the endosomal membranes of cells, rather than on plasma membranes; hence, ligand binding by the LRR motifs of these TLRs occurs in the lumen of intracellular vesicles. It is generally accepted that the extracellular nucleic acids released from damaged tissues or cells, infected or uninfected, are endocytosed and presented to the internal TLRs. Alternatively, nucleic acids from bacteria or viruses, which multiply within a cell, can be captured in membranous vesicles and then transported to TLRs in the endosomes. Activation of these TLRs leads to the induction of interferons, proinflammatory cytokines, and chemokines. Cell-surface TLRs also sense endogenous ligands, released in damaged tissue as a danger signal, resulting in the induction of inflammation under both infectious and noninfectious conditions. In a number of recent studies, TLRs have been found on a wide range of cells, including immune cells such as mast cells, macrophages, and dendritic cells. TLRs are also found on epithelial cells and mesenchymal fibroblasts, and these cells recognize microbial infections by sampling the exterior milieu using a group of receptors that are able to discriminate between potential pathogens and self-produced molecules.

## 4. Expression and Function of TLRs in the Human Female Genital Tract

### 4.1. TLR1, TLR2, and TLR6

TLR2 is structurally related to TLR1 and TLR6 [38]. TLR2 forms heterodimers with TLR1 and TLR6, which is involved in discriminating between the molecular structures of diacyl and triacyl lipopeptides [39–41]. Complexes of TLR1 and TLR2 recognize various microbial components, such as lipoproteins/lipopeptides, lipoarabinomannan, and PGNs from gram-positive and gram-negative bacteria and mycoplasma [14, 42, 43]. It also recognizes lipoteichoic acid from gram-positive bacteria, a phenol-soluble medulin from *Staphylococcus aureus*, glycolipids from *Treponema maltophilum* [14, 44], GPI anchors of protozoa [45], and zymosan and phospholipomannan from fungi [46, 47]. TLR2 is also reported to be involved in the recognition of atypical LPS from nonenterobacteria, the structures of which are different from the typical LPS of gram-negative bacteria [44, 48]. Experimentally, TLR2 has been shown to recognize synthetic lipoproteins [Pam_3_Cys-Ser-(Lys)_4_] [40]. During microbial infection in the female genital tract, TLR2 is considered to recognize the PGN of *C. trachomatis* [42, 49, 50], LPS and fragments of PGN of *Neisseria gonorrhoeae* [51–53], and phospholipomannan of *Candida albicans* [54]. TLR2 is also involved in the recognition of viral components such as cytomegalovirus and herpes simplex virus type 1 [55–57]. CD36, a member of the class II scavenger family of proteins, was shown to serve as a facilitator or coreceptor for diacyl lipopeptide recognition through the TLR2/6 complex [58]. Both TLR2 and TLR6 are necessary for responding to mycoplasma-associated protein. 

Constitutive TLR1 and TLR6 expression has been detected in the epithelial cells of the fallopian tubes, endometrium, endocervix, ectocervix, and vagina [59–67]. TLR1 expression has also been detected in uterine NK cells [68], vascular endothelial cells, and smooth muscle cells within the stroma of the cervix and the myometrial smooth muscle cells of the uterus [63]. Whereas, TLR6 expression was detected in uterine NK cells [51] and in stromal fibroblasts within the vagina [63]. 

Constitutive expression of TLR2 has been reported in the epithelial cells of the fallopian tubes, endometrium, cervix, and vagina [59, 60, 62–66, 69, 70], smooth muscle cells of the cervix and vagina [63, 71], endometrial stromal cells [70], and uterine NK cells [68, 72]. The highest levels of TLR2 mRNA expression have been observed in the fallopian tubes and cervical tissues, followed by the endometrium and ectocervix [60]. The expression levels of TLR2 in endometrial stromal cells were comparable to those of endometrial epithelial cells. Significantly higher levels of expression of TLR2 and TLR6 in the endometrium have been observed during the secretory phase than in other phases of the menstrual cycle [56, 67, 73]. TNF-*α* upregulates the TLR2 expression in human cervical smooth muscle cells [71].

 Pam_3_Cys-Ser-(Lys)_4_, a synthetic analog of bacterial lipopeptides that bind to TLR2/1 heterodimers, was found to induce the production of MIP-3*α* and TNF-*α* by endometrial epithelial cells [74]. Polyriboinosinic : polyribocytidylic acid [poly (I : C)], a TLR3 agonist, induced the expression of TLR2 in human fallopian tube epithelial cells [65]. FLS-1, a TLR2/6 heterodimer agonist, induced the expression of proinflammatory cytokines and chemokines in the epithelial cells of cervix and vagina [66]. Lipoteichoic acid inhibits human cytomegalovirus infection in ectocervical tissue through induction of IFN-*β* production [75]. *Mycoplasma genitalium *and the C-terminal portion of the antigenic protein encoded by MG309 activate NF-*κ*B via TLR2/6, resulting in cytokine secretion from the epithelial cells of the uterine cervix and vagina [37]. Whereas, polyanionic microbicides, such as dextran sulfate and polystyrene sulfonate inhibits TLR1/2- and TLR2/6-mediated cytokine production by human cervical and vaginal epithelial cells [76]. TGF-*β* is reported to inhibit the TLR-2-mediated activation of uterine NK cells [72].

### 4.2. TLR3

TLR3 recognizes dsRNA and is considered to mediate various antiviral responses. dsRNA during viral infection can arise from several sources [25]. The genome of the infecting virion can itself be dsRNA, as in the case of the known natural dsRNA viruses. However, even ssRNA virus samples often contain defective particles contain primarily double-stranded defective genomes. Intracellular viral dsRNA can be generated in a number of ways. In the case of ssRNA viruses, the formation of dsRNA replication intermediates is an obligatory step in viral reproduction. In the case of DNA viruses, complementary mRNAs are often produced that are encoded by partially overlapping genes located on the opposite strands of the viral genome. Long viral polycistronic mRNAs often contain abundant stable double-stranded stems. Such findings, taken together, have indicated that all viral infections induce dsRNA at some point during replication [77]. Recently, host-derived mRNA released by dying or dead cells was shown to activate TLR3, suggesting that activation via TLR3 can occur in a variety of situations [78]. It has been demonstrated that a secondary structure creating hairpin loops within the mRNA is responsible for TLR3 activation. In addition, TLR3 has been shown to recognize double-stranded nucleic acid from *Schistosoma mansoni* and to be involved in the antiparasite response [79]. At present, it is generally accepted that RNA from a number of different sources can activate TLR3, as long as the RNA displays a secondary structure containing double-stranded regions, provided that the RNA is present in the appropriate cellular vesicle.

In experimental models, poly (I : C), a synthetic analog of viral dsRNA, is utilized as a ligand for TLR3 to mimic viral infection [18, 22, 65, 80, 81]. The induction of TLR3 signaling via dsRNA activates transcription factors such as NF-*κ*B and IRF3, resulting in the production of proinflammatory and antiviral cytokines and chemokines [18, 82–84]. In the clinical setting, a variety of viruses (e.g., herpes simplex virus, human papilloma virus, hepatitis B virus, hepatitis C virus, cytomegalovirus, human immunodeficiency virus, etc.) can be the causative of viral infection in the female genital tract. 

Constitutive expression of TLR3 has been reported in female genital tissue samples from fallopian tubes, endometrium, cervix, and vagina [60, 65, 66]. The expression of TLR3 in the endometrium is significantly higher during the secretory phase than in other phases of the menstrual cycle [67, 83]. TLR3 expression has been detected in the epithelial cells of the fallopian tubes, endometrium, endocervix, ectocervix, and vagina [59, 62–65, 70, 84–87]. TLR3 expression was also detected in endometrial stromal cells, although expression levels were higher in endometrial epithelial cells than in endometrial stromal cells [70]. Jorgenson et al. [85] recently demonstrated the cycle-dependent expression of TLR3 in primary endometrial epithelial cells. TLR3 expression was also detected in the stromal fibroblasts of the vagina and endocervix [63], and in human uterine NK cells [68, 72].

Poly (I : C), a TLR3 agonist, induces the expression of proinflammatory cytokines, chemokines, and TLR3 in human fallopian tube epithelial cells [65, 87]. Cultured fallopian tube epithelial cells recognize viral dsRNA via TLR3 and secrete proinflammatory cytokines and chemokines via an NF-*κ*B-mediated signal pathway [87]. Poly (I : C) alone does not stimulate IFN-*γ* production by IL-2-expanded uterine NK cells [88]. Whereas, the presence of autologous uterine macrophages led to a significant increase in IFN-*γ* production by IL-2-expanded uterine NK cells [88]. Whereas, polyanionic microbicides, such as dextran sulfate and polystyrene sulfonate, inhibit TLR3-mediated cytokine production by human cervical and vaginal epithelial cells [76].

It has been demonstrated that human endometrial epithelial cells recognize dsRNA and produce proinflammatory cytokines and chemokines via a TLR3-mediated pathway [64, 85, 89]. Lesmeister et al. [90] showed that in vitro treatment of endometrial epithelial cell lines with 17*β*-estradiol had no effect on TLR3 expression, and treatment with 17*β*-estradiol suppressed the production of proinflammatory cytokines and chemokines resulting from TLR3 stimulation with poly (I : C); these findings suggest that 17*β*-estradiol modulates TLR3 function. Poly (I : C) also upregulated the production of IL-8 by the epithelial cells of the uterine cervix [66, 86] and the production of proinflammatory cytokines and chemokines by the epithelial cells of the vagina [66]. In addition, Poly (I : C) activated these cells and induced IFN-*γ* production [75]. TGF-*β* was shown to inhibit the TLR-3-mediated activation of uterine NK cells [91]. Poly (I : C) inhibits human cytomegalovirus infection in ectocervical tissue through induction of IFN-*β* production [75].

### 4.3. TLR4

LPS is a cell-wall component of gram-negative bacteria. LPS is composed of lipid A (endotoxin), core oligosaccharide, and O-antigen. TLR4 recognizes lipid A of LPS. In addition to bacterial LPS, TLR4 also recognizes heat-shock protein 60, glycoinositolphospholipids of protozoa [45], and viral envelope proteins [92–94]. The ligation of TLR4, in association with the accessory molecules MD-2 and CD14, leads to the recruitment of MyD88, the phosphorylation of IL-1 receptor-associated kinase, the oligomerization of TNF receptor-associated factor 6, and the subsequent degradation of I*κ*B [30, 31]. These events lead to the activation of NF- *κ*B, and to the resultant transcription of immune response genes, such as proinflammatory cytokines, chemokines, and costimulatory molecules, which are necessary for further immune responses [8, 95, 96]. The host response to a primary bacterial infection of a mucosal surface is acute inflammation and is characterized by the infiltration of neutrophils and monocytes. In the clinical setting, a variety of microorganism-derived substances including LPS derived from *N. gonorrhoeae* [51–53], LPS and heat shock protein derived from *C. trachomatis* [97, 98], and mannan derived from *C. albicans* [54] are putative ligands for TLR4 in the female genital tract. 

Constitutive expression of TLR4 was reported in the following female genital tissues: the fallopian tubes, the endometrium, the cervix, and the vagina [60, 66]. TLR4 expression has been shown to decline progressively along the genital tract, with the highest levels of expression in the fallopian tubes and endometrium, followed by the cervix [60]. The expression of TLR4 in the endometrium is significantly higher during the secretory phase compared with that in other phases of the menstrual cycle [67, 83]. Conflicting findings regarding the expression of TLR4 in the epithelial cells of the female genital tract have been reported. Some authors' groups have reported the presence of TLR4 in the epithelial cells of the fallopian tubes [63, 65], endometrium [62–64, 70, 99], endocervix [63, 66], and vagina [57, 66]. However, other authors have observed an absence of TLR4 in the epithelial cells of the fallopian tubes [100], endocervix [59], ectocervix [59, 63], and vagina [59, 63]. The expression of TLR4 has also been detected in endometrial stromal cells [70], myometrial cells [101], uterine NK cells [68, 72], and smooth muscle cells of uterine cervix [71]. Levels of expression of TLR4 were found to be higher in endometrial stromal cells than in endometrial epithelial cells [70].

CD14, a coreceptor of TLR4 for the recognition of LPS, is not expressed in human fallopian tube epithelial cells or stromal cells [100]. However, CD14 has been detected in endometrial stromal fibroblasts, although it was not found in endometrial epithelial cells [99]. Whereas, Herbst-Kralovetz et al. [66] demonstrated the expression of CD14 in the epithelial cells of cervix and vagina. CD14 is known to be expressed in human cervical smooth muscle cells [71]. MD2, an accessory molecule of TLR4-signaling, was found to be absent from cultured epithelial cells derived from samples of normal human vagina, endocervix, and ectocervix [59]. 

 Binding to LPS, a TLR4 ligand, rapidly leads to NF-*κ*B activation and cytokine expression via TLR4-mediated signaling in fallopian tube stromal fibroblasts [100]. However, fallopian tube epithelial cells that lack TLR4 do not respond to LPS. LPS was shown to stimulate the expression of IL-8 in endometrial epithelial cells and stromal fibroblasts via a TLR4-mediated pathway [99]. LPS also induced the production of MIP-3*α* in primary endometrial epithelial cells [74], but not in an endometrial epithelial cell line, HHUA [102]. It has been demonstrated that cultured endocervical epithelial cells were unresponsive to LPS from either *N. gonorrhoeae* or *Escherichia coli* [59]. LPS induced the translocation of the NF-*κ*B p65 subunit in human myometrial cells via a TLR4-protein kinase *ζ*-mediated pathway [101]. IFN-*γ* was also found to enhance the expression of TLR4, CD14, MD2, and MyD88 in endometrial stromal fibroblasts [99]. LPS inhibits human cytomegalovirus infection in ectocervical tissue through induction of IFN-*β* production [75].

### 4.4. TLR5

TLR5 recognizes flagellin, a protein component of bacterial flagella [15]. It has been suggested that TLR5 serves as a sensor for pathogenic bacteria that is able to cross the epithelium [103]. 

TLR5 expression has been demonstrated in epithelial cells derived from the fallopian tubes, endometrium, vagina, endocervix, and ectocervix [59–65]. TLR5 expression was also detected in smooth muscle cells and vascular endothelial cells within the stroma of the vagina and endocervix [63]. However, TLR5 expression was not detected in human uterine NK cells [68]. The expression of TLR5 in the endometrium is significantly higher during the secretory phase than during other phases of the menstrual cycle [67, 83]. 

Flagellin, a TLR5 agonist, induced the expression of proinflammatory cytokines and chemokines in the epithelial cells of cervix and vagina [66].

### 4.5. TLR7

TLR7 has been shown to recognize self- and guanosine- or uridine-rich viral ssRNA from viruses such as HIV, vesicular stomatitis virus, and influenza virus [19, 20]. TLR7 signaling is also induced by low molecular-weight antiviral compounds, that is, imidazoquinolines [104].

TLR7 expression has been detected in the epithelial cells of fallopian tubes, endometrium, cervix, and vagina [61, 65, 66, 84, 105]. TLR7 expression was also detected in uterine NK cells [68] and in the endometrial stroma [69]. 

Poly (I : C), a TLR3 agonist, was shown to induce the expression of TLR7 in human fallopian tube epithelial cells [65]. Imiquimod, a TLR7 agonist, was demonstrated to stimulate IL-8 production by the primary cultured cells isolated from fallopian tube, endometrium, and cervix [105].

### 4.6. TLR8

TLR8 has been shown to recognize both self- and guanosine- or uridine-rich viral ssRNA from viruses such as HIV, vesicular stomatitis virus, and influenza virus [19, 20, 106]. 

TLR8 is expressed in the epithelial cells of fallopian tubes, endometrium, cervix, and vagina [61, 63–65, 73, 86, 105]. TLR8 expression was also detected in the endometrial stroma, as determined by immunohistochemical analysis [73]. However, no TLR8 expression has been detected in human uterine NK cells [66]. CL075, a TLR8 agonist, was demonstrated to stimulate IL-8 production by the primary cultured cells isolated from fallopian tube, endometrium, and cervix [105].

### 4.7. TLR9

TLR9 recognizes DNA containing unmethylated deoxytidyl-phosphate-deoxyguanosine (CpG) motifs common to both bacterial and viral genomes [17, 104, 107]. CpG motifs are found in the genomes of DNA viruses such as herpes simplex virus [108–110], suggesting that TLR9 induces antiviral responses in herpes genitalis. TLR9 also recognizes nonDNA pathogenic components, such as hemozoin and genomic DNA derived from protozoa malarial parasites [43, 111]. Experimentally, TLR9 signaling has also been induced by synthetic CpG-rich oligonucleotides (CpG-ODN) [17]. 

TLR9 expression has been reported in fallopian tube, endometrium, and cervix [105]. TLR9 expression has been demonstrated in epithelial cells of the fallopian tube, endometrium, cervix, and vagina [61, 62, 64–66, 70, 73, 86]. TLR9 expression was also detected in endometrial stromal cells [70, 73]. The authors of the latter study noted observing comparable expression levels of TLR9 in endometrial epithelial cells and stromal cells. However, higher levels of expression of TLR9 in the endometrium have been reported during the secretory phase than during other phases of the menstrual cycle [67, 73]. Moreover, TLR9 expression was absent in human uterine NK cells [68]. 

 CpG oligodinucleotides upregulated the production of IL-8 by the epithelial cells of the fallopian tube and uterine cervix [65, 86]. CpG oligodinucleotides, a TLR9 agonist, was demonstrated to stimulate IL-8 production by the primary cultured cells isolated from fallopian tube, endometrium, and cervix [105]. To date, there have been no studies of TLR9 expression in the vaginal mucosa. CpG oligodinucleotides inhibit human cytomegalovirus infection in ectocervical tissue through induction of IFN-*β* production [75].

### 4.8. TLR10

A specific ligand for TLR10 has yet to be identified [23, 112, 113]. TLR10 expression has been demonstrated in fallopian tube, but not in endometrium or cervix [105]. TLR10 expression is absent in human endometrial epithelial cells and endometrial epithelial cell lines [60, 64]. However, Aflatoonian et al. [73] demonstrated using immunohistochemistry that TLR10 is expressed in the endometrial epithelium and stroma. The same authors also reported that levels of expression of TLR10 in the endometrium are significantly higher during the secretory phase than during other phases of the menstrual cycle [73]. TLR10 expression has also been detected in human uterine NK cells [68]. TLR10 has not been detected in human fallopian tube epithelial cells [65]. Higher levels of expression of TLR9 in the endometrium have been reported during the secretory phase than during the proliferative phase of the menstrual cycle [67].

## 5. Conclusions

It has been suggested that there is a site-specific mucosal immune system in the female upper genital tract that differs from that described in the gastrointestinal and respiratory tracts [114]. Furthermore, the immune system in the upper genital tract differs from that of the lower genital tract. The putative immune system in the upper genital tract appears to contribute to the maintenance of an aseptic milieu; that is, this immune system inhibits the growth of microorganisms that sporadically colonize this region [4]. In contrast, the lower genital tract is constantly exposed to microorganisms, including species of commensal as well as pathogenic organisms; in general, abundant anaerobic bacterial flora is known to be present in the vagina. The mucosal components of the lower genital tracts have adapted to a dynamic, nonsterile environment challenged by a variety of antigenic/inflammatory stimuli associated with sexual intercourse and endogenous vaginal microbiota. Clearly, it is essential that these mucosal tissues develop mechanisms for selectively responding to pathogens, while simultaneously avoiding chronic inflammation due to immune responses to commensal microorganisms [5]. The sequelae of a chronic inflammation in the female genital tract would be highly detrimental to the host and would include increased transmission of sexually transmitted diseases [6].

As summarized in this paper, the innate immune system in the female genital tract is highly complex and multifactorial. Mucosal epithelial cells, fibroblasts, lymphocytes, macrophages, and dendritic cells associated with the female genital tract have evolved a unique mechanism for the recognition of pathogens. These cells express a variety of TLRs, allowing them to recognize the different repertoire of a wide range of PAMPs. It is likely that TLR distribution in the female genital tract reflects an immunological tolerance of commensal organisms in the lower portions of the tract (i.e., vagina, ectocervix, and to some extent, the endocervix), as well as an intolerance of commensal microbial flora in the upper portion of the tract (i.e., the endometrium and fallopian tubes). The mucosal surface of the upper portion of the female genital tract is generally considered a sterile site, in part due to the cervical mucus, which filters bacteria and other debris. However, this barrier can readily be crossed by a variety of infectious agents, typically leading to endometritis and salpingitis. Thus, it is essential that the upper genital tract epithelium has the capacity to recognize and respond to ascending pathogens while simultaneously avoiding a state of unnecessary inflammation that might disrupt the epithelial barrier. The sequelae of such inflammation in the upper genital tract would be highly detrimental to the defense and reproductive functions of the mucosal surface. If the luminal epithelial barrier is broken by acute inflammation, damaged epithelial cells initiate and coordinate the inflammatory response, alerting adjacent epithelium and underlying immune cells of the potential danger posed by various microorganisms. 

 STDs are a major worldwide health problem that compromise reproductive fecundity and cut short the lives of millions of men, women, and children [115, 116]. Despite extensive efforts, only limited success has been achieved in dealing with STDs such as *N. gonorrhoeae*, *C. trachomatis*, group B streptococcus, herpes simplex virus type 2, and HIV. These pathogenic organisms can be recognized by TLRs expressed in the female genital tract. Further investigation into TLR signaling in these tissues could provide new insights into the roles played by the immune system in maintaining health and combating STDs and other genital infectious diseases.

## Figures and Tables

**Table 1:** Human TLRs and their cognate ligands.

TLRs	Ligand
TLR1	triacyl lipopeptides, modulin (bacteria)
	Pam_3_Cys-Ser-(Lys)_4_ (synthetic lipoprotein)
TLR2	peptidoglycan, lipoprotein, lipopeptides, atypical LPS,
	lipoteichoic acid, phenol-soluble modulin (bacteria)
	zymozan, lipoarabinomannan (fungi)
	GPI anchors, glycolipids (protozoa)
	envelope protein (virus)
	Pam_3_Cys-Ser-(Lys)_4_ (synthetic lipoprotein)
TLR3	dsRNA (virus)
	mRNA (host)
	poly (I : C) (synthetic dsRNA)
TLR4	LPS, lipoteichoid acid, mannuronic acid polymers (bacteria)
	mannan, glucoronoxylomannan (fungi)
	heat-shock protein 60, glycoinositolphospholipids (protozoa)
	envelope protein, F protein (virus)
	heat-shock protein 60, heat-shock protein 70, polysaccharide fragments of heparin sulfate, hyaluronic acid, fibrinogen, fibronectin DA domain (host)
TLR5	flagellin (bacteria)
TLR6	diacyl lipopeptide, modulin, soluble tuberculosis factor (bacteria)
TLR7	ssRNA (virus)
	ssRNA (host)
	imidazoquinoline (synthetic antiviral compound)
	loxoribine (guanosine analog)
TLR8	ssRNA (virus)
	ssRNA (host)
TLR9	unmethylated CpG DNA (bacteria, protozoa, virus)
	hemozoin (protozoa)
	CpG-ODN (synthetic CpG-rich oligonucleotide)
	Chromatin-IgG complex (host)
TLR10	Unknown
